# Home‐Based Falls Intervention for People with Dementia: Insights from a Process Evaluation

**DOI:** 10.1002/alz70858_099063

**Published:** 2025-12-24

**Authors:** Leanne Greene, Louise M Allan, Bethany Whale, Rowan H Harwood, Vicki M Goodwin, Obioha Ukoumunne, James Connors, Adam Gordon, Thomas Jackson, Rachael Litherland, Sarah Morgan‐Trimmer, Claire M Hulme, Abigail Hall, Robert Barber, Steve Parry, Simon Conroy, Chris Fox, Ashima Sharma, Alison Bingham

**Affiliations:** ^1^ University of Exeter, Exeter, Devon, United Kingdom; ^2^ University of Nottingham, Nottingham, Nottinghamshire, United Kingdom; ^3^ University of Birmingham, Birmingham, West Midlands, United Kingdom; ^4^ Innovations in Dementia CIC, Exeter, United Kingdom; ^5^ University of Exeter, Exeter, United Kingdom; ^6^ Northumberland, Tyne and Wear NHS Foundation Trust, Newcastle Upon Tyne, Tyne and Wear, United Kingdom; ^7^ Newcastle Hospitals NHS Foundation Trust, Newcastle, Tyne and Wear, United Kingdom; ^8^ Central and North West London, London, London, United Kingdom

## Abstract

**Background:**

Falls among people with dementia often result in physical and psychological repercussions, leading to reduced independence and increased economic burden on healthcare systems. Although this population faces heightened fall risks, robust evidence for effective home‐based interventions remains scarce.

**Method:**

This study used a multiple‐methods process evaluation as part of a pilot cluster randomised controlled trial, guided by a realist approach. The study was conducted across six UK sites (three intervention and three control). Fidelity assessments were conducted on routine data collection, intervention delivery, multidisciplinary team (MDT) meetings, and supervision sessions. Semi‐structured interviews were carried out with people with dementia, caregivers, and therapists delivering the intervention.

**Result:**

The Maintain intervention achieved high fidelity in home assessments and intervention implementation, with participants attending an average of 15 of the 22 planned sessions. Qualitative analysis indicated that regular home visits enhanced engagement and motivation. MDT support boosted therapists' confidence, particularly in addressing complex cases. While most participants felt they met their functional goals and reported improved confidence, challenges included geographic variability in service delivery due to staff capacity, fear of secondary falls, and inconsistent referral pathways. Therapists’ perceptions of advanced dementia influenced the intervention's execution. The dyadic approach encouraged activity engagement but occasionally increased caregiver responsibilities.

**Conclusion:**

The Maintain intervention was feasible and well‐received, showing potential for improving daily living activities and quality of life in people with dementia. A future trial should focus on standardising MDT support, incorporating strategies to address falls‐related anxiety, and developing sustainable post‐intervention maintenance plans. Adaptations, such as video consultations, may mitigate workforce constraints and enhance accessibility.

